# Characterization of FDG PET Images Using Texture Analysis in Tumors of the Gastro-Intestinal Tract: A Review

**DOI:** 10.3390/biomedicines8090304

**Published:** 2020-08-24

**Authors:** Anne-Leen Deleu, Machaba Junior Sathekge, Alex Maes, Bart De Spiegeleer, Mike Sathekge, Christophe Van de Wiele

**Affiliations:** 1 Department of Nuclear Medicine, AZ Groeninge, 8500 Kortrijk, Belgium; anneleen.delue@azgroeninge.be (A.-L.D.); alex/maes@azgroeninge.be (A.M.); 2 Department of Nuclear Medicine, University of Pretoria, 0002 Pretoria, South Africa; chabi.sathekge@gmail.com (M.J.S.); mike.sathekge@up.ac.za (M.S.); 3 Department of Morphology and Medical Imaging, University Hospital Leuven, 3000 Leuven, Belgium; 4 Department of Analytical Chemistry, DruQuaR, University of Ghent, 9000 Ghent, Belgium; bart.despiegeleer@ugent.be; 5 Department of Diagnostic Sciences, University of Ghent, 9000 Ghent, Belgium

**Keywords:** radiomics, gastro-intestinal cancer

## Abstract

Radiomics or textural feature extraction obtained from positron emission tomography (PET) images through complex mathematical models of the spatial relationship between multiple image voxels is currently emerging as a new tool for assessing intra-tumoral heterogeneity in medical imaging. In this paper, available literature on texture analysis using FDG PET imaging in patients suffering from tumors of the gastro-intestinal tract is reviewed. While texture analysis of FDG PET images appears clinically promising, due to the lack of technical specifications, a large variability in the implemented methodology used for texture analysis and lack of statistical robustness, at present, no firm conclusions can be drawn regarding the predictive or prognostic value of FDG PET texture analysis derived indices in patients suffering from gastro-enterologic tumors. In order to move forward in this field, a harmonized image acquisition and processing protocol as well as a harmonized protocol for texture analysis of tumor volumes, allowing multi-center studies excluding statistical biases should be considered. Furthermore, the complementary and additional value of CT-imaging, as part of the PET/CT imaging technique, warrants exploration.

## 1. Introduction

FDG PET imaging is a well-established imaging modality for staging, restaging, and follow-up of a wide variety of human malignancies. Tumor uptake of FDG as assessed by FDG PET imaging is commonly quantified by the semi-quantitative standardized uptake value (SUV) and the maximum (SUVmax) and mean SUV (SUVmean) values of tumor uptake have been frequently adopted in clinical trials and clinical routine [1]. However, reported changes in SUVmax/mean and sometimes SUVmax/mean pre- or post-treatment alone derived from FDG-PET imaging prior to or following treatment were shown to be conflicting in terms of their potential to predict pathologic response and long-term prognosis in a wide variety of cancers, with an overall reported low accuracy [2,3]. Factors potentially responsible for this low accuracy reported include inconsistent-cut-off values for discriminating benign from malignant activity, increase in SUVmax values in secondary inflammatory processes following chemotherapy and radiotherapy, early SUVmax reduction in the presence of viable tumor tissue, a spatially heterogeneous response, partial volume effects, and change in body composition and habitus [4]. Accordingly, other predictive and prognostic parameters than SUVmax values, derived from FDG PET imaging, allowing a more accurate prediction or stratification of responders and non-responders to cancer treatment, are of major clinical interest. 

A substantial spatial heterogeneity in tumor cellular and molecular characteristics, many of which have been shown to contribute to FDG-uptake, has been reported previously [5]. The most extensively documented tumor characteristics in this regard include cellular proliferation, differences in blood flow and neo-angiogenesis, oxygenation, and gene expression [4,5,6]. Several of these factors have been independently associated with a more aggressive behavior, a poorer treatment response and a worse prognosis. Intra-tumor heterogeneity of FDG-uptake can potentially be quantified with textural features extracted from obtained PET images through complex mathematical models of the spatial relationship between multiple image voxels. Accordingly, texture analysis is emerging as a new tool for assessing intra-tumoral heterogeneity in medical imaging. Here, we review available literature on texture analysis using FDG PET imaging in patients suffering from tumors of the gastro-intestinal tract. 

## 2. Eligible Studies

In this review, we only included studies that provided original data, were designed to provide information on response to tumor treatment, prognosis or biological tumor characterization, and were conducted in patients suffering from known malignancies of the gastro-intestinal tract. Both prospective or retrospective designed as well as blinded or non-blinded studies were considered for inclusion. Case-control studies, case-reports, and case-series were excluded. We searched the databases of Medline and Embase for studies published throughout 2020 using a combination of medical subject headings terms and free text words to define our patient population of interest (patients suffering from gastro-intestinal tract malignancies) and both the specific imaging technique (18F-FDG PET/CT) and image analysis tool (radiomics or texture analysis). Two reviewers (A.D.L and C.V.d.W) independently screened all retrieved studies as well as the references included in the retrieved articles seeking any additional potentially relevant articles based on their title and abstract and subsequently evaluated the full text of the selected articles for eligibility. Studies were included following discussion and by consensus.

## 3. Technical Aspects

### 3.1. Tumor Volume Segmentation and Volume Requirements

Various methods for tumor segmentation have been used for the purpose of texture analysis, these include manual delineation, semiautomatic delineation applying either a threshold of the maximum value of the tumor, and region growing, most often 40%, or a fixed SUV cut-off value, most often 2.5 or 3.0 SUV, as well as gradient based fully automated segmentation (e.g., FLAB) [7,8]. For obvious reasons, manual delineation suffers from high inter-observer and intra-observer variability. On the other hand, thresholding, either as a percentage of the SUV-max or using an SUV cut-off inherently excludes intra-tumoral regions with low or no uptake e.g., areas of necrosis, thus reducing tumor heterogeneity. Inversely, gradient-based methods e.g., Fuzzy logic Bayesian Approach Tool, include the entire tumor, including the areas without uptake. As the contour defined by the latter software algorithms is binary, only they are theoretically to be preferred over the manual and semi-automated methodologies. 

### 3.2. Volumetrics

In terms of volumetrics, the minimum tumor volume required for adequate intensity sampling has been estimated to be about 700 voxels based on the fact that statistics derived from FDG PET images are a comparison of intensity distributions that are to be compared via a chi-square goodness –of-fit test [9]. When using voxels with a planar and trans-axial resolution of 4 mm (0.064 cm^3^ per voxel) as customary for most FDG PET acquisitions, this corresponds to a volume of 45 cm^3^ or a tumor with a diameter of 4.5 cm. On the other hand, texture analysis of clinical data in a series of 555 patients suggests that texture analysis may add valuable complementary information for tumor volumes above 10 cm^3^ [10]. 

### 3.3. SUV Discretization or Resampling Image Intensity Values

The purpose of SUV discretization is to reduce the otherwise infinite possible number of intensity values to a finite set as well as effectively reducing image noise. Possible options for SUV discretization include using a fixed number of discrete bins (e.g., 16 or 32) dividing the image SUV range into equally spaced intervals which will result in discretized images with varying bin sizes or ‘intensity resolution’ depending on the SUV range of the tumors studied, termed lesion relative sampling [11,12]. More specifically, the SUV range for each lesion will be scaled differently meaning that the same bin number in two different lesions will not correspond to the same original SUV values [13]. Alternatively, fixed bin sizes may be used e.g., 0.5, without (lesion absolute resampling, the first and last bin being determined individually for each lesion) or with discretization of the whole SUV-scale which allows for maintaining a constant intensity resolution or SUV scale across all tumor images e.g., 0 to 30, termed absolute resampling [11,14]. 

### 3.4. Textural Feature Extraction

Textural parameters derived from tumors visualized using FDG-PET imaging are mostly obtained through statistics-based techniques, based on the spatial distribution of voxel values, calculating local features at each pixel in the image and deriving parameters from the distributions of the local feature. [4,5,15]. The statistical methods are categorized into first-order (one voxel), second-order (two voxels) and higher-order (three or more voxels) statistics. First-order parameters are histogram analysis based and include mean, minimum, and maximum intensity, standard deviation, skewness, and kurtosis. Second-order features, e.g., entropy, energy, contrast homogeneity, and dissimilarity are calculated using spatial grey level dependence or co-occurrence matrices that determine how often a pixel of intensity finds itself within a certain relationship to another pixel of intensity j. Finally, local higher order parameters such as coarseness, busyness, and complexity can be derived from neighborhood grey-tone difference matrices, whereas regional parameters can be derived from voxel alignment matrices e.g., run-length emphasis and variability or from grey-level size zone matrices e.g., zone emphasis and size-zone variability.

## 4. Esophageal Carcinoma

Both chemotherapy and chemoradiotherapy have been adopted in the neoadjuvant armamemtarium of potentially curative esophageal cancer, mainly based on the MAGIC-, OEO2-, and CROSS-trials, showing a 5-year OS-advantage in respectively 13%, 6%, and 14% of patients [16,17,18]. A pathologic complete response (pCR) to chemoradiotherapy (CRT) is observed in approximately 25–30% of esophageal cancer patients. A reliable prediction of pCR before surgery would enable investigators to study the feasibility and outcome of an organ-preserving strategy after chemoradiotherapy that includes omission of surgery, associated with a high morbidity and mortality rate, and close clinical follow-up allowing for a more personalized treatment approach. To date, both endoscopic biopsies, qualitative EUS (endo-echoscopic ultra sound), and qualititative as well as quantitative FDG PET have been assessed for their potential to detect residual disease following CRT. A recent systematic review and meta-analysis by Eyck et al. suggest an insufficient accuracy of all these modalities for detecting residual disease as evidenced by their pooled sensitivities and specificities, respectively 33% and 95% for endoscopic biopsies, 96% and 8% for qualitative EUS, 74% and 52% for qualitative PET, 69% and 72% for PET-SUVmax, and 73% and 63% for PET %Delta-SUVmax [19]. Accordingly, more powerful predictors of pCR are of major clinical interest. In this regard, several authors have assessed the added predictive value of texture analysis of FDG-PET images for pCr when compared to the classical SUV-values (see Table 1). Tixier et al. performed texture analysis on pre-treatment FDG PET images obtained in 41 patients suffering from esophageal carcinoma that underwent combined radiochemotherapy and found that the best AUC-values for separating complete responders form partial -or non-reponders using receiver operating curve (ROC)-analysis were intensity variability, size-zone variability, entropy, and local homogeneity [13]. Sensitivity and specifity obtained using these variables for separating complete responders from partial- and non-responders proved significantly larger than those obtained using SUV analysis. As part of a validation study on 555 patients by Hatt et al., the pre-treatment FDG PET images of 112 esophageal carcinoma patients were analyzed using texture analysis [10]. When dichotomized with optimal cut-off values in the Kaplan–Meier analysis, both dissimilarity and metabolic tumor volume could differentiate survival curves. Tan et al. studied twenty patients suffering from esophageal cancer that underwent CRT followed by surgery [20]. In all patients, FDG PET/CT imaging before and after CRT was available. Pre- and post FDG PET/CT images were rigidly registered, tumor-volumes semiautomaticllay delineated using an SUV threshold ≥2.5 followed by manual editing and comprehensive features extracted to characterize the SUV intensity distribution, texture patterns, tumor geometry, and associated changes resulting from CRT using the ITK open source software. While the best traditional response measure was SUVmax decline (AUC 0.76), SUVmean decline and skewness as well as the texture features inertia, correlation, and cluster prominence also proved significant predictors of pCR with AUC-values of respectively 0.79, 0.76, 0.85, 0.80, and 0.78. Van Rossum et al. studied 217 esophageal adenocarcinoma patients that underwent CRT followed by surgery, 59 of which were shown to have a cPR, and in whom a baseline FDG-PET/CT and a post CRT FDG PET/CT examination were available [21]. Using a semi-automated gradient-based tumor delineation method followed by manual editing, a large variety of tumor textural features were derived using an in-house developed software. Aside from post-CRT TLG, the texture features baseline cluster shade, delta run percentage, Delta ICM entropy, and post CRT roundness proved significant predictors of pCR in multivariate analysis. However, when included in a predictive model, the gain in AUC as compared to FDG PET/CT based subjective assessment of responses proved minimal, respectively 0.72 versus 0.77, and insufficient to base the decision to omit surgery upon (decision threshold ≥0.9). As rigid registration of images from different treatment time points as performed in the series by Tan et al. may be inadequate to account for tumor deformation, propagation of tumor contour between longitudinal PET/CT images using image registration of their CT-counterparts may provide an automatic way to re-contour tumor volumes for textural features computation. In this regard, Yip et al. compared the values of textural features derived from ten deformable registration algorithms versus those obtained by rigid registration in a series of 45 esophageal cancer tumors treated by CRT that underwent subsequent surgery and in whom pre- and end-CRT PET/CT images were available. [22] It was found that fast-demons, fast-free-form, and rigid algorithms should be applied with care due to their inherent performance compared to optical flow algorithms (Lucas–Kanade, Horn–Schunck, Least mean Square, Iterative, Fast iterative, and Inverse consistency Horn–Schunck (IHS)). Of the three textural features studied, respectively gray level co-occurrence matrix derived entropy, run length matrix derived short-run high gray run emphasis and size zone matrix derived short-zone high gray emphasis, only the two former yielded significant AUC values (>0.70) with IHS yielding systematically the highest value of 0.78 for both textural features. As opposed to the study by Yip et al., rather than selecting a limited number of textural features ab initio, in a study on 65 patients suffering from esophageal carcinoma treated by CRT, Desbordes et al. studied 61 initial textural features and defined the best subset of complementary features using a random forest classifier [23]. Furthermore, these authors compared the best predictive and prognostic subset of features to those obtained by a Mann–Whitney study (predictive study) and a univariate Kaplan–Meier analysis (prognostic study). Out of the 28 features that were not correlated, the ones that predicted best complete response to therapy were metabolic tumor volume and homogeneity from the co-occurrence matrix (respective AUC values of 0.84 and 0.81). The best prognostic subset found was composed of MTV, WHO status, and nutritional risk index. More recently, Foley et al. reported on a prognostic model identifying increased log total lesion glycolysis (TLG) and histogram kurtosis and reduced log (histogram energy) as parameters being independently associated with worse overall survival in a cohort of 403 oesophageal carcinoma patients, 302 of which formed the development cohort, whereas the remaining 103 patients formed the validation cohort [24]. Of interest, in this study, the best fitting PET automatic segmentation method was selected in each individual case from a range of available segmentation methods including adaptive thresholding, Fuzzy C-means and region growing methods. As FDG PET/MRI imaging may provide an opportunity to improve phenotyping by combing molecular, functional and anatomic characteristics, Baiocco et al. explored whether combined FDG PET/MRI radiomics proved different between oesophageal carcinoma patients presenting with and without distant metastases in a series of 20 patients, ten of which presented with distant metastases [25]. In their preliminary series, high ADC entropy combined with low SUV entropy were associated with a higher prevalence of metastases and a promising initial signature for future study. Finally, as opposed to the above studies, in a series of 52 esophageal cancer patients by Nakajo et al., texture analysis performed on the pre-treatment FDG PET images had limited value in prediction of prognosis of patients with esophageal cancer treated by chemoradiotherapy in multivariate analysis [26].

## 5. Gastric Carcinoma

Several large-scale randomized controlled studies have shown the beneficial effects of adjuvant chemotherapy in reducing or delaying relapse following initial curative surgery for gastric cancer [27]. However, in spite of adjuvant chemotherapy being administered, the survival rates for many patients, regardless of initial high response rates, remain low. Given that the current TNM classification does not provide full prognostic information in this regard, additional parameters that can be used to better predict patient outcomes and chemotherapy responses are of interest. In this regard, Jiang et al. retrospectively studied the radiomic signature of FDG PET baseline imaging for prediction of gastric cancer survival and chemotherapeutic benefits in 214 gastric carcinoma patients, 132 of which formed the training cohort and 82 the validation cohort [28]. Tumor contours were manually delineated, the SUV image was discretized by a 0.1 SUV unit bin width and a total of 80 quantitative features were extracted from each volume of interest of each patient’s PET image to characterize intratumor heterogeneity and complexity. Incorporating a radiomic score derived from the training cohort, separating patients in a low and a high-risk group, into a radiomics based nomogram resulted in better performance than TNM staging and the clinicopathologic nomogram. Furthermore, patients presenting with higher radiomic scores were prone to benefit from chemotherapy.

## 6. Hepatocellular Carcinoma (HCC)

Imaging has proven essential to guide therapy in patients suffering from HCC, the second most common cause of cancer mortality worldwide. A number of studies have previously identified a correlation between SUV values of primary HCCs and outcomes following different systemic and locoregional treatment strategies, including selective internal radiation therapy (SIRT) with 90Y-labbeled microspheres [29]. In a recent study by Bland-Durand et al., whole-liver radiomics was used to create a scoring system to predict PFS and OS, classifying HCC patients into a low- and a high-risk subgroup, in a retrospective cohort of 47 unresectable HCC patients undergoing Yttrium-90 radioembolization [30]. The radscore classifications proved significantly associated with PFS and OS in the multivariate analysis and its prognostic value did not differ when stratified by the Barcelona-Clinic Liver Cancer staging system or tumor size. The authors suggested their model incorporated metabolic liver function in addition to tumor biology, which has been shown to influence HCC prognosis.

## 7. Pancreas Carcinoma

Despite advances in surgery, RT, and CHT, prognosis of patients suffering from ductal pancreatic ductal adenocarcinoma (PDAC) remains poor with a 5-year survival inferior to 25% [31]. Accordingly, novel prognostic biomarkers aside from the classical TNM staging system identifying high-risk patients, requiring a more aggressive treatment, may impact treatment management and ultimately also patient outcome. Thus, Hyun et al. assessed retrospectively whether intratumoral heterogeneity measured by PET texture analysis has potential as a prognostic imaging biomarker in a series of 137 patients suffering from newly diagnosed PDAC. Using a gradient-based segmentation method, a resampling to 64 discrete bins and the open-source software package Chang-Gung Image Texture Analysis Toolbox, 4 first-order and 27 higher-order textural features were extracted from the primary tumor metabolic volume defined on the staging PET/CT examination [32]. Values obtained were related to overall survival. In multivariate Cox analysis, after adjusting for age, gender, clinical stage, tumor size and CA 19-9 level, only tumor entropy proved associated with worse survival (*p* = 0.028, AUC = 0.72). Inversely, in a series by Yue et al. including 26 PDCA patients, higher order textural features proved not significantly related to OS. The authors used the clinical planning target volume to extract a VOI for texture analysis and resampling to an unspecified finite range of gray levels. Finally, Cui et al. retrospectively studied 139 patients suffering from locally advance pancreatic cancer, 90 of which formed the training cohort and 49 of which the validation cohort, which were treated with stereotactic body radiation therapy. In their series, tumors were manually delineated, and the SUV histogram comprised between the 2.5% and 97.5% quantiles was divided into 32 equal bins [33]. Subsequently, 173 image features were extracted of which seven image features were finally selected using an elastic net-regularized Cox regression model based on the training cohort. When tested retrospectively on the training cohort, the proposed signature provided a higher 95% CI score of 0.62 when compared to conventional imaging indicators, including tumor volume, SUVmax, and TLG (95% CI, 0.57–0.58).

## 8. Colorectal Carcinoma

While overall mortality of colorectal carcinoma has decreased by almost 50% compared to its historical peak in 1980, it remains high in locally advanced rectal cancer (LARC) with a 5-year mortality rate around 30% stressing the need for the identification of patients that may benefit from more aggressive treatment and follow-up [34,35,36]. Following standard treatment of LARC, involving surgical resection preceded by neoadjuvant CRT or RT alone, pCR is reached in approximately 15–30% of patients. In these patients, a reliable prediction of pCR before surgery based on FDG-PET tumor texture analysis might also enable investigators to study the feasibility and outcome of an organ-preserving strategy after chemoradiotherapy and to allow for a more personalized treatment approach (see Table 2). Nakajo et al. studied 32 patients presenting with newly diagnosed colorectal carcinoma and assessed the potential of texture analysis of FDG PET images to predict progression free survival. Tumor boundaries were derived using an SUV-threshold of 2.5 and intensity rescaled using 64 discrete values. Texture parameters studied included intensity variablity (IV), size-zone variability (SZV), zone percentage (ZP), and coefficient of variation (COV). Texture analysis was only performed if the MTV exceeded 10 cm^3^ [37]. At bivariate analysis, aside from tumor stage, IV (*p* = 0.004) and SZV (*p* = 0.028) proved significantly related to progression free survival. Lovinfosse et al. retrospectively studied 86 patients with LARC (stage III rectal carcinoma treated by neoadjuvant CRT [38]. Of the texture parameters extracted from tumor volumes derived using the fuzzy locally adaptive Bayesian (FLAB) algorithm and a linear quantization into 64-gray levels, homogeneity and coarseness were significantly associated with disease free survival, whereas SUV mean dissimilarity and contrast from the neighborhood intensity-difference matrix were significantly and independently associated with overall survival in multivariate analysis. Giannini et al. studied 57 colorectal LARC patients, 42 of which received CRT and 15 of which received RT with a simultaneous integrated boost, that had underwent both FDG PET/CT imaging and MRI imaging prior to initiation of their neoadjuvant treatment [39]. All patients underwent surgery at the end of their treatment, twenty-two patients were classified as responders (nine with TRG = 1 and 13 with TRG = 2 (Mandard’s five-point assessment scheme)) and the remaining thirty patients as non-responders. Five patients were excluded due to MRI artefacts or because the TRG score was not evaluated. Segmentation of tumors on PET images was obtained using an automatic adaptive threshold algorithm, whereas in MRI images, tumors were segmented using an in-house developed algorithm using C++ and the ITK libraries. When combining texture features extracted from both PET and MRI images, a model was generated including PET homogeneity, PET contrast, PET 10t% quantile, glycolytic volume, metabolic volume, and T2w correlation, yielding an AUC value of 0.86, while sensitivity and specificity using the point on the ROC-curve yielding the maximum value of the Youden index (sensitivity+ specificity-1) as cut-off point, respectively 0.42, were 86% and 83%.

Lovinfosse et al. also retrospectively looked at the relationship between parameters derived from texture analysis of FDG PET imaging and biological characteristics of 151 newly diagnosed primary colorectal carcinoma and found that SUVmax, mean, standard deviation, and coefficient of variation as well as skewness proved significantly associated with the presence of RAS mutations (*p* values ranging between 0.049 and 0.001) [40]. However, related AUC-values for predicting the presence of RAS-mutations proved ≤0.65 limiting the clinical value of these parameters as predictors for K-ras mutation. Importantly, this study included all ranges of primary tumor sizes (from T1 to T4). Finally, Rahmin et al. assessed the prognostic value of FDG PET radiomic features in 52 patients with colorectal intra-hepatic only metastases and found that, in addition to the number of liver-metastases and metabolic tumor volume, additional measures of intra-tumor heterogeneity derived from texture analysis resulted in further enhanced prediction of OS and PFS when included in the multivariate prognostic model [41].

## 9. Discussion

Variables derived from texture analysis of FDG-PET images in patients suffering from tumors of the gasto-intestinal tract predictive for treatment outcome were shown to vary widely from one study to another in the same cancer type (see Table 1 and Table 2). Various factors may have contributed to this finding.

First, in many of the studies reported, acquisition and reconstruction parameters were not reported and thus may have differed significantly. It has been previously shown that radiomic features are sensitive to FDG PET acquisition and reconstruction parameters. In a series of 20 patients with lung lesions on FDG PET, out of 55 texture features and six features based on first-order statistics, iteration number and full-width at half maximum were shown to have a significant impact on texture features [42]. The features that displayed the smallest coefficient of variation were entropy, difference entropy, inverse difference normalized, inverse difference moment normalized, low gray-level run emphasis, high gray-level emphasis and low gray-level zone emphasis. Adopting a similar methodology in a study of twenty patients suffering from solid tumors by Glaavis et al., textural features with a low coefficient of variation were entropy-first order, energy, maximal correlation coefficient, and low-gray level run emphasis due to different acquisition models and reconstruction parameters [43]. A harmonization method, termed Combat, that involves removing the center effect while preserving patient-specific effects, and standardized textural features derived from PET images obtained using different imaging protocols, was recently proposed by Orlhac et al. [44]. The method proved easy to use, to retain biological effects not related to a center effect, and did not require any feature recalculation. The method was suggested to allow for multicenter studies and for the external validation of radiomic models or cutoffs and to facilitate the use of radiomic models in clinical practice.

Second, in the series included in this review, various methodologies were used for tumor delineation, respectively automated gradient-based methods, 40% threshold region growing methods and using a 2.5 SUV-cut-off. The choice of an SUV-cut-off of 2.5 is based on early studies demonstrating that this cut-off is optimal for differentiating benign from malignant lesions and minimizes unwanted physiological uptake in normal tissues, whereas a fixed threshold of 40% was shown to best approximate tumor volume [45,46,47,48,49,50,51,52]. While the gradient based methods are theoretically the preferred ones, as they allow assessment of the entire tumor, including areas of necrosis, they are not widely available and currently their use is limited to those research centers where they were developed [53,54]. Furthermore, limited available data show that these techniques provide similar information to that obtained using threshold techniques and that, when performed on EARL-compliant PSF images, they provide an accurate means of overcoming reconstruction variability in metabolic tumor volume delineation [55]. In addition, while voxel dimensions were usually 4 × 4 × 4 mm, in some studies, voxel sizes of 4 × 2 × 2 mm were used. When using smaller voxels, the same uptake pattern is seen as more homogenous. In this regard, using simulated spheres, Orlhac et al. demonstrated that homogeneity and long run emphasis were the most variable with voxel size, with an increase of 35.5% and 85.5%, respectively, between a sphere described with voxels of 2 and 4 mm, whereas entropy and short-run emphasis were less influenced and low-grey level zone emphasis and high grey-zone level emphasis were robust to voxel size [56].

In the series reported in this review, the majority of studies did not report on the way in which SUV values were discretized. However, the manner of SUV discretization was shown to have a crucial effect on the resulting textural features and the interpretation thereof, emphasizing the need for a standardized methodology in tumor texture analysis. As shown by Leijenaar et al., discretizing using a fixed number of bins or discrete resampling values is less appropriate for inter- and intra-patient comparison of textural values in a clinical setting [11]. The interpretation of textural features using discrete resampling or a fixed bin size, or energy resolution, is overall different between both discretization methods and, for several features, affected by the choice of intensity resolution.

The assumed minimal volume required for proper texture analysis reported in literature varies from 45 cm^3^, based on statistical considerations, to 10 cm^3^ based on assessment of the complementary nature of texture analysis, and functional tumor volume in a multi-cancer site cohort of 555 patients by Hatt et al. [9,10]. As shown in the latter study, the smaller the tumor volume, the less complementary parameters derived from texture analysis are. In the majority of studies included in this review, tumor volumes studied were not systematically reported, yet, based on the above, such information appears vital in order to be able to draw correct conclusions as to the general validity of the results reported. For instance, drawing general conclusions on the usefulness of texture analysis based on a study including predominantly small tumor volumes would lead by definition to an underestimation of the predictive value of texture features. Furthermore, in the series included in this review, the performance of texture indices was only rarely compared to the performance of metabolic tumor volume, a variable to which many of the texture indices have been shown to be significantly correlated. This makes it difficult to assess the added value of texture analysis to MTV.

Most of the studies included in this review have reported on a small number of patients and identified multiple image-derived texture features with no pre-specified analytical model which may have resulted in a statistical type-I error inflation. In a study by Chalkidou et al., applying appropriate statistical corrections on a series of 15 published studies dealing with texture analysis of PET and CT studies in oncology, an average type-I error probability of 76% (range: 34–99%) was estimated with the majority of published results not reaching statistical significance [57]. Furthermore, out of these 15 studies, only three used a validation dataset. Likewise, in the studies reported in this review paper, few studies included a testing set and a validation set. In addition, several studies have shown that many PET texture features are highly correlated with each other and with tumor volume, a collinearity which may lead to instability of the regression coefficients weights in a multivariate model with small changes in the data leading to very different regression coefficients. While some studies reported in this review corrected for this phenomenon better known as "the bouncing betas", this was not the case for all studies reported in this review. Both phenomena could explain in part why in a similar patient population e.g., colorectal or esophageal carcinoma, texture features identified and/or cut-off values of prognostic significance differ from one study to another in this review.

In terms of clinical relevance, AUC-values obtained using ROC-analysis in those studies that found texture analysis derived indices to be predictive for treatment outcome did not reach 0.9, a requirement for individual clinical applicability, thus limiting their clinical usefulness.

Finally, with the exception of one study, respectively by Lovinfosse et al. [40], none of the other studies included in this review made an attempt was to explore the relationship between radiomic features and the underlying tumor biology. However, the link between metabolic, genomic, histologic, clinical, and imaging parameters is essential in order to establish an effective personalized and reliable treatment strategy, especially when confronted with limited available tissue for analysis [58].

While it would have been interesting to combine all the above series in a meta-analysis for the different oncological entities studied, for all of the above reasons, such a meta-analysis was impossible.

In-vivo quantitative information, including texture-analysis, of neoplastic processes of the gastro-intestinal tract may also be derived from images obtained using current state-of the-art CT and MRI imaging [59,60]. Their added clinical value to in-vivo quantitative information derived from FDG/PET images warrants exploration.

In conclusion, due to the lack of technical specifications, a large variability in the implemented methodology used for texture analysis, and lack of statistical robustness, currently, no firm conclusions can be drawn regarding the predictive or prognostic value of FDG PET texture analysis derived indices in patients suffering from gastro-enterologic tumors. In order to move forward in this field, a harmonized image acquisition and processing protocol as well as a harmonized protocol for texture analysis of tumor volumes, allowing multi-center studies excluding statistical biases, should be considered. Furthermore, the complementary and additional value of CT-imaging, as part of the PET/CT imaging technique, warrants exploration.

## Figures and Tables

**Table 1:** Studies investigating texture analysis in esophageal carcinoma patients.

Authors	Nb pts	Camera	Volume Segmentation	MTV	Clinically Relevant Variables
Tixier et al. 2011 [13]	20	NS	GBM	NS	entropy, local homogeneity, intensity variability, size-zone variability
Tan et al. 2013 [20]	20	Philips	SUV ≥ 2.5	NS	inertia, cluster prominence, correlation
Hatt et al. 2015 [10]	129	Philips	GBM	specified for the entire cohort of 555 patients	when dichotomized with optimal cut-off values in the KM analysis; MTV and dissimilarity
Yip et al. 2015 [22]	45	GE	>40% RG	6–440 cm^3^	entropy, short high grey run emphasis
Van Rossum et al. 2016 [21]	27	NS	GBM	NS	post-treatment TLG
Desbordes et al. 2017 [23]	65	Siemens	GBM	2.5–141 cm^3^	MTV, homogeneity
Nakajo et al. 2017 [26]	52	GE	SUV ≥ 2.5	10.2–282 cm^3^	MTV, TLG, intensity variability, size-zone variability
Foley et al. 2018 [24]	403-202 development set-101 validation set	GE	in-house segmentation method	NS	log TLG, log histogram entropy, log histogram kurtosis

Nb = number, NS = not specified, GBM = gradient based method, GE = General Electric, SUV = standardized uptake value, MTV = metabolic tumor volume, TLG = total lesion glycolysis, KM = Kaplan–Meier survival analysis.

**Table 2:** Studies investigating texture analysis in colorectal carcinoma patients.

Authors	Nb pts	Camera	Volume Segmentation	MTV	Clinically Relevant Variables
Nakajo et al. 2017 [37]	32	GE	SUV ≥ 2.5	10.1–120 cm^3^	heterogeneity parameters, intensity variability, size-zone variability
Lovinfosse et al. 2017 [38]	86	Philips	GBM	NS	SUVmax, dissimilarity and contrast
Giannini et al. 2019 [39]	57	Philips	adjusted threshold algorythm	NS	MTV, TLG, homogeneity, contrast

Nb = number, NS = not specified, GBM = gradient based method, GE = General Electric, SUV = standardized uptake value, MTV = metabolic tumor volume, TLG = total lesion glycolysis.
